# The science underlying frugal innovations should not be frugal

**DOI:** 10.1098/rsos.180421

**Published:** 2019-05-22

**Authors:** Balkrishna C. Rao

**Affiliations:** Department of Engineering Design, Indian Institute of Technology Madras, Chennai 600036, India

**Keywords:** factor of safety, factor of frugality, advanced frugal innovation, sustainable development

## Abstract

In recent years, *frugal* products of the grassroots and advanced types are being widely used due to their *sustainable* nature and also affordability. However, despite the origins of *frugality* at grassroots level, grassroots innovations continue to be fabricated in an ad hoc manner that precludes application of science and are thus susceptible to premature failure. This work advocates the use of scientific principles for developing *frugal* products in general with emphasis on *classical* and new design methodologies that are rooted in science to save resources, and hence lower costs, while aiming for robust product functionality. This paper sheds light on the importance of the *safety factor* in *frugal* designs and the need, from here onwards, of a *factor of frugality* for the systematic realization of both grassroots and *advanced frugal* products. In particular, adoption of the *factor of frugality*, that was developed recently, has been supported in this effort with a numerical example to display the effectiveness of applying science for designing from scratch *frugal products* that are both streamlined and robust in their functionality.

## 1. Introduction

*Frugal innovations* [1] or innovations resulting from doing-more-with-less [2] have become widely popular due to their no-frills nature that entails lower costs. These innovations with their genesis at a grassroots level [3,4] in the emerging markets of China and India are now being increasingly sought in sectors involving sophisticated technology. The quintessential grassroots *frugal innovations* fall under *Jugaad*, which are makeshift contraptions for executing routine chores [5]. In recent years, several grassroots innovations, such as MittiCool^®^ [6], have been reported that provide ingenious solutions to problems in various settings against a natural scarcity in resources including raw material, knowledge, skilled workforce, supply chains, to name a few. Grassroots *innovations* in emerging markets are therefore typically created under poorer conditions that are in stark contrast to innovating under conditions of rich nations clubbed under the *Organisation for Economic Co-operation and Development* (OECD).

In recent years, frugality has also been adopted in innovations requiring sophisticated *science and technology* (S&T). Such *frugal* products have been reported [7] under a new class called *advanced frugal innovations* (AFI), which use scientific principles and, in some cases, cutting-edge research for their realization. As opposed to their grassroots brethren, AFIs can be created by chasing frugality in a rich setting that is actually commensurate to innovating under conditions of ample resources. In other words, AFIs are realized by intentionally setting constraints on the amount of resources consumed in realizing an innovation and as such can be followed by public and private entities of rich and emerging nations alike. Therefore, AFIs are designed against an artificial constraint of scarcity in resources as opposed to the grassroots type, whose design is constrained by a poorer background characterized by natural resource scarcity. These AFIs have appeared in a range of sectors including teaching, healthcare, automotive and aerospace, to name a few [1,7].

The minimalist nature of these innovations endears them to the cause of sustainability for tackling planetary crises such as climate change and resource scarcity while uplifting the living standards of society at large [1,7,8]. However, most of the *frugal innovations*, at least at the grassroots level, are makeshift contraptions, made from indigenous ingenuity, that achieve their goals under natural constraints on various resources. Consequently, it is important that sound scientific principles are either not overlooked or haphazardly applied in realizing these innovations irrespective of their grassroots or sophisticated nature. Such scientific grounding would aid in improving the potential of these innovations that could be harnessed for uplifting society at large.

This paper therefore argues on the need to use science, sometimes at the cutting edge, to realize grassroots and *advanced frugal innovations* that are not prone to failure under normal working conditions and also marginal conditions of overloading. The use of the *factor of frugality* as the *new safety factor* in product design is advocated in this work, whose utilization will allow the systematic design from scratch of grassroots innovations and AFIs. This paper starts by invoking the sustainability side of *frugal innovations* that make it imperative to involve science in their designs. This is followed by sections stressing the use of scientific principles of design including the *factor of frugality,* an extension of the *safety factor*, for making *frugal* products robust and safe.

## 2. Frugal innovations and sustainability

The continued progression of both *climate change* and resource scarcity has made it indispensable to leave a minimal carbon footprint in all activities of every nation on this planet. Moreover, planetary and man-made crises, such as the recent financial meltdown of 2008–2009 [9], are encouraging the development of low-cost products and low-cost services that are affordable to society at large for improving their living standards [10]. These twin challenges of affordability and sustainability can be tackled by the widespread usage of *frugal* products and *frugal* services. This is because, other than their lower costs, *frugal innovations* also possess green credentials due to their no-frills nature that entails lesser consumption of resources and hence generally lesser emissions [11], which are both beneficial to the environment.

However, the successful realization of *frugal innovations* against the above backdrop is significantly dependent on the systematic application of scientific principles found in product design. In other words, achieving a low-cost product with minimal resources that has robust functionality needs the sound application of design methodologies that are deeply rooted in science. This is because the fabrication of a given part with minimal material resources typically makes it ‘flimsy’ i.e. susceptible to failure under nominal and even moderate conditions of abnormal loadings, an instance of overload failure due to insufficient material has been reported by de Faria *et al*. [12] in railroads. Therefore, the traditional or *classical factor of safety* approach to design should be extended, as in a recently developed method [13], to the *factor of frugality* approach for systematically creating, from scratch, streamlined products possessing robust functionality.

The design and development of AFIs reported by Rao [1,7] are already rooted in the application of science. Examples of such AFIs are the portable ECG and particle accelerator that can be realized only through detailed studies and cutting-edge research in the areas of biomedical engineering and theoretical-and-experimental aspects of particle physics, respectively. Although, all of the *frugal innovations* listed in [7] and a majority of those outlined in [1] have their genesis in science, tools for the methodical creation of *frugal innovations* from scratch have been developed only recently [13].

## 3. Factor of safety

The *classical* approach to design is dependent on the *factor of safety* (*N*), which is given by

3.1 $$N=\frac{\sigma_{M}}{\sigma_{W}},$$

where *N* is defined as the ratio of maximum material stress (*σ*_M_), such as yield, to the actual working stress (*σ*_W_). The failure condition typically refers to permanent deformation due to plastic yielding, which will impair product functioning and hence is associated with the failure of the product concerned. Therefore, failure stress or *σ*_M_ is taken to be the yield strength of a product's material.

## 4. Frugal design approach

This paper focuses on the design aspects of *frugal innovations* and as such will deal only with the minimization of consumption in material resources. This enables the creation of *frugal* products from scratch (or at the conceptual stage) by directly influencing the materials and manufacturing aspects of design. Frugality in using other resources such as supply chains and labour for lowering costs are considered secondary in importance to the methodical achievement of frugality directly in products.

Therefore, the *safety factor* can be extended to a *factor of frugality* (*F^N^*) [13], whose use in design facilitates the utmost frugality in resource consumption while realizing the product. The symbolic representation of *F^N^* comprises the *factor of frugality* (*F*) and the *safety factor* defined by equation (3.1). The *frugality factor* is in turn given by

4.1 $$F=N\;+\;MS_{1}\;+\;MS_{2}\;+\;MS_{3}\;+\;MS_{4}\;+\;MS_{5},$$

where *N* has a low value that is slightly in excess of 1—to avoid working at the failure load—and the various *material saved* (MS) parameters with indices from 1 to 5 refer to the quantification of material savings achieved in schemes such as a simple design, apt materials, apt manufacturing techniques, biomimetics or nature-based solutions, and salvaging from *end of life* (EOL) systems, respectively. An *EOL* system refers to any product at the end of its life and hence heading towards the ‘junkyard’. It should be noted that MS parameters have been isolated to account for individual design aspects only for clarity in explaining the concept of *frugality factor*. Some of these parameters could be clubbed together in real design, such as simple design and materials.

Therefore, starting with an *N* of 1.5, the designer sifts through extraneous *material-saving* schemes to conserve material in excess of savings coming from a low *N* value. In other words, non-dimensional ratios or fractions of weight of material saved through MS schemes, typically between ‘bulky’ and ‘lean’ versions, are added to the *factor of safety*, which is also a number, to arrive at a numerical value for *F*. It is important to work on MS schemes in the following sequence while computing *F*: simple design; materials; manufacturing, biomimetics and salvaging. The representation *F^N^* literally extends the *factor of safety* and also facilitates standardizing a maximum value for *F*, for a given set of MS schemes, against which the *F* value for the given design can be compared to determine its efficacy [13]. Consequently, a higher *F* value for a given low value of *N* signifies an improvement in the streamlining of the concerned product.

**Figure 1.**
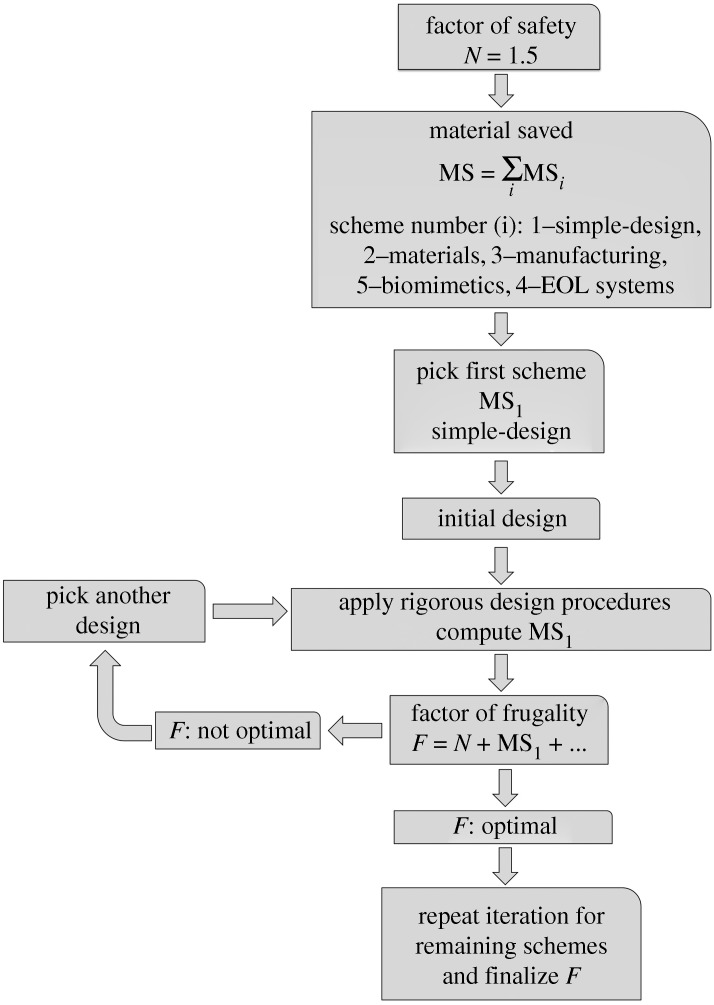
Flowchart depicting iterative nature of a typical material-saving scheme such as simple design. Details have been avoided for clarity in understanding and also brevity.

The *frugal design* approach should be carried out iteratively against the entire gamut of various *material-saving* schemes until a proper *F* value is arrived at for the given product. These iterations will facilitate convergence to a proper mix of MS values corresponding to apt extraneous material-saving schemes. Figure 1 depicts a flowchart showing an instance of the iterative nature of the *frugal* approach where different designs for a product at a fixed *N* value have to be attempted before arriving at a maximum value for MS_1_ and hence *F*. Although this charts the flow for only the first MS scheme, i.e. simple design, subsequent schemes will follow the same flow in arriving at the final value for *F.*

Moreover, in many cases, only a subset of the relevant MS schemes will have to be added for a given design, with the model represented by equation (4.1) allowing ease of defining and adding MS factors corresponding to other material-saving routes that are not described here. Although newer design techniques employing big data such as topology optimization [14] could be applied individually, their integration into the *factor of frugality* approach would facilitate both quantification of frugality and avail extra savings of material through the involvement of MS schemes such as salvaging from EOL systems.

## 5. Results

### 5.1. Classical approach with factor of safety

A lower value of *N* typically results in a design operating closer to the failure condition with *N* = 1 denoting working stress equated to the failure stress. As a result, the use of a low *N* value should be accompanied by rigorous design procedures as in the aircraft industry where an *N* value of 1.3–1.5 [15–17] is typically used for improving fuel efficiency. However, a lower *factor of safety* in design also gives the benefit of consumption of lesser amounts of materials for the realization of a product. *Frugal innovations*, either of the grassroots or advanced type with a no-frills structure, typically consume minimal amounts of material and hence fall into the low *safety factor* category and could thus be vulnerable to failure under nominal and/or even marginal conditions of overloading.

Traditionally, an *N* value much higher than 1.3–1.5 is assumed when the designer perceives uncertainties in the numerous inputs going into the design. These inputs comprise analytical process models; material behaviour models; and manufacturing, to name a few [13]. In other words, the product is ‘padded’ with extra material to offset any failures arising from these uncertain sources.

An instance of designing with a lower *N* value can be demonstrated with a basic element of engineering design such as a shaft, which is the workhorse for transmitting power and/or conversion of rotary motion to linear and vice versa. The shaft has also been selected because its design brings out the features critical to robust functioning of *frugal* products in general. Table 1 lists results from the design of a shaft with extrema in *N* of 1.5 and 3. The details of designing this shaft under torsional loads for transmitting a power of 500 kW at 1200 r.p.m. can be found in Ugural [18]. The results listed in table 1 clearly show that savings in weight are accompanied by reduced margins for overloading as *N* goes down to a value of 1.5. The overloading entries are calculated from the baseline and maximum torque, which correspond to the basic design and torque resulting in incipient plastic deformation, respectively.

**Table 1.** *Classical* approach to designing a shaft (material: steel, yield shear strength = 300 MPa and shear modulus = 80 GPa).

no	safety factor *N*	savings in weight (%)	baseline torque (N m)	maximum torque (N m)	overload %
1	1.5	37	3979	5970	50
2	3	—	3979	11 940	200

### 5.2. Frugal approach to design

**Figure 2.**
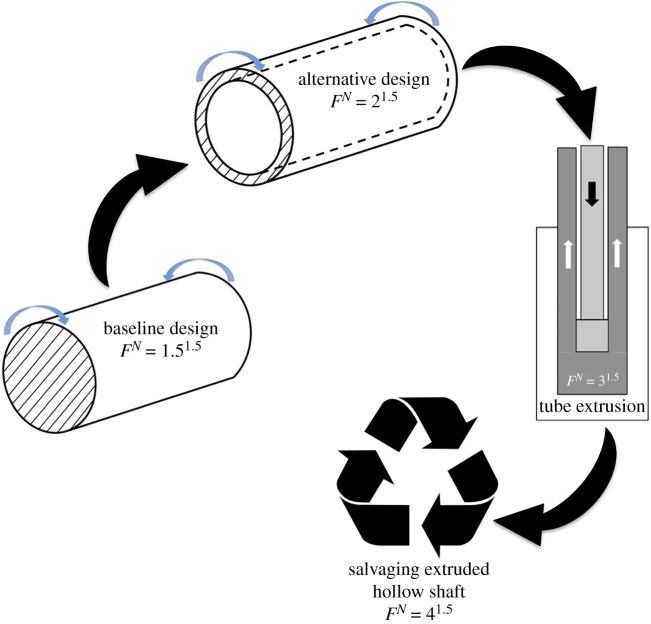
Schematic showing cumulative improvement in the *factor of frugality* of a shaft transmitting power.

Table 2 lists results of designing the shaft through *frugal approach* that employs the *factor of frugality* without iterations. The first entry corresponds to *classical* design of a solid shaft (first entry of table 1) whose *factor of frugality* is same as the *N* of 1.5. The second entry lists results of the *frugal approach* for an *N* of 1.5 where there are extra savings from selecting a hollow shaft by *classical* design; extrusion as opposed to machining of such a hollow shaft; and salvaging such an extruded hollow shaft from an EOL system; have been quantified and added according to equation (4.1) to obtain an *F^N^* of *4^1.5^*. The design details of these entries can be found in [13]. It should be noted that the baseline case could be the hollow shaft itself but starting with a solid shaft covers the general case and also brings out the additional weight savings realized by opting for a hollow part. Moreover, the *frugal approach* should be applied to different designs for a given product and the optimal design would be the one with the highest *F* value for the given application (figure 1). Only three of the five MS factors in equation (4.1) are used here because of their relevance to shaft design. Table 2 brings out the maximum savings in material possible for the shaft by combining the low *safety factor* design with additional savings realizable through apt schemes for this *classical* design. Figure 2 illustrates the various steps involved in achieving a *factor of frugality* of *4^1.5^* for the shaft.

**Table 2.** *Frugal* approach to shaft design.

no	design approach	safety factor *N*	material-saving schemes			factor of frugality *F^N^*	overload %
			alternative design MS_1_ (hollow shaft)	manufacturing technique MS_3_ (extrusion)	salvaging from EOL system MS_5_		
1	*Baseline*	1.5	—	—	—	1.5^1.5^	50
2	*Frugal*	1.5	0.5	1	1	4^1.5^	50

## 6. Discussion

A *factor of safety* of 3 in table 1 results in a design that can handle an overload—in excess of rated torque—of 200% vis-à-vis an overload of 50% tolerated by the baseline design with *N* of 1.5. The amount of overload that can be withstood by a design becomes even lower for *N* tending closer to unit value with *N* = 1 signifying incipient plastic deformation. Therefore, such weaker margins in overloads for a very low *N* would possibly lead to the early onset of failure in the shaft and *frugal* products in general. However, the design for *N* *=* 3 uses materials in excess by weight of 37% when compared to baseline.

*Frugal innovations* fall in the category of a low *safety factor* design wherein material savings are achieved at the expense of vulnerability to failure under various conditions of loading. Such vulnerability can impair the functionality of *frugal* products, irrespective of the grassroots or advanced types, with serious consequences especially in critical sectors such as healthcare. In fact, grassroots innovations could be vulnerable even within the domain of nominal working conditions due to their makeshift nature that excludes the application of any design methodologies. In particular, any of the following, and other reasons, individually or in combination, could lead to premature failure of a makeshift *frugal innovation*: intuitive economizing of material usage that pays no scientific attention to weak spots; impromptu medley of EOL parts, whose integrity individually has not been vetted; and improper assembly of parts, to name a few.

However, a low *N* design is used to advantage in the *frugal design approach*. The lower *factor of safety* is used as the first step in this approach reported recently by Rao [13]. An *N* value of 1.5 should be selected for this purpose with the attendant rigorous design procedures, as followed by the aircraft industry [15–17]. The rigour in design is warranted to both avoid material-padding to offset design-related uncertainties and achieve robust functionality under nominal working conditions with a possibility of even sustaining some overloads—as inferred from the design showcased in table 1 where the shaft is capable of sustaining a 50% excess torsional load. It should be noted that the *frugal* hollow shaft is also capable of sustaining a 50% overload. Other than sustaining overloads, an instance of a frugally designed product having better load bearing abilities vis-à-vis *classical* design is evidenced by the hollow shaft in table 2 that has improved sectional properties [19] for enhanced structural rigidity when compared to a solid shaft designed under the same set of conditions including an *N* value of 1.5. Therefore, products scientifically designed by the *frugal approach* are not necessarily *frugal* in their performance. In addition to economizing from the baseline step of the *frugal approach*, other schemes rooted in *sustainability* are also used to skim as much material as possible for a given product. In the shaft example covered in this work, these schemes involved the use of the hollow feature, as an alternative design, to minimize consumption of raw materials; manufacturing the hollow shaft with reduced waste; and salvaging such an extruded hollow shaft. Overall, the use of these extraneous material-saving schemes on top of a low *N* design has improved the *factor of frugality* by 167% in going from 1.5 to 4.

The *frugal design approach* presented by Rao [13] can also be applied to other basic elements of engineering design such as plates, beams, springs, etc. Each of these frugally designed components will typically be assembled into a *frugal* product (or *frugal innovation*). It should be noted that loads arising from the interaction between individual *frugal* parts after assembly should be foreseen by the designer and used as commensurate constraints on the concerned parts during their *frugal* design.

Although the *factor of safety* approach can also be applied to designing such innovations, the *frugal* approach brings out the additional material-saving schemes that are not accounted for by the *classical* design process. In particular, material saved through schemes such as salvaging from EOL systems and use of an apt manufacturing technique are extraneous and hence completely absent in the *classical* design approach. Ideally, the *factor of frugality* sums up savings from all possible sources to truly quantify the *frugal* use of materials while aiming for the robust functionality of a design.

Even though *classical* design principles have been used in arriving at *F^N^*, the *frugality factor* has been established to formally encourage and also ease the adoption of green practices by *frugal* and non-*frugal* designers alike for all-round *sustainable development.* Therefore, low *safety factor* coupled with rigorous design; extraneous material-saving schemes and low cost are significant features of *frugal design* that would beckon designers and engineers to go *frugal* especially when humankind needs to simultaneously fight *climate change* and uplift living standards of society at large. In this regard, equation (4.1) can be applied to systematically build *frugal* products from scratch and also attribute an *F^N^* score to rank their credentials for *frugality* or judicious use of resources. Such ranking and subsequent labelling of products with *F^N^* scores would, as a tool of *ecodesign,* facilitate efforts for progressing in all-round *sustainable development.*

The urgency to improve our environment through widespread *sustainable development* is warranted due to the following reasons. First, *climate change* is making it incumbent upon us to significantly lower our carbon footprint so as to leave a better planet to posterity. Second, the swelling ranks of people in lower- and middle-income segments around the world are aspiring for better living standards [20] that are also affordable. In this regard, systematically designed *frugal innovations* offer the right mix of lower carbon footprint, robust functionality and lower price. However, excluding science by employing makeshift techniques to fabricate these innovations could result in premature product failure that can impede the diffusion of these innovations and hence impair their significant potential for *sustainable development*. It should be noted that the *factor of frugality*, as given by equation (4.1), is built to account for *frugal* features originally seen in grassroots innovations and later observed in advanced products. Therefore, applying the *frugal design approach* to a grassroots concept will lead to a successful and systematic scientific realization of a grassroots innovation. Other than grassroots ingenuity leading to the concept of *frugal design*, this is all the more important due to the significant impact grassroots innovations can make to the rich and poor alike in a *sustainable* economy when systematically designed and fabricated by leveraging scientific principles. The *factor of frugality* therefore is an apt *ecodesign* [21,22] measure for facilitating maximum savings in material resources and also quantifying it for any innovation qualifying as *frugal*.

Although creators of AFIs can readily adopt the *frugal design approach* due to their possession of requisite wherewithal, grassroots innovators typically lack the skills and also resources to do so. Therefore, governmental and relevant non-profit entities should set up subsidized programmes to empower grassroots innovators with science including *frugal design* through academicians, scientists and other qualified workers. Public entities should shoulder the main responsibility of creating awareness and also informing *frugal* innovators in general about relevant institutions and associated scientific workers to contact for seeking design solutions. These services including actual design work should be offered free of cost to grassroots innovators. The private sector should also be encouraged to select and support, in terms of funding and knowledge, design work for promising grassroots innovations that could possess the potential for commercial success. Overall, this work urges involvement of public, private and other relevant entities in educating, funding and empowering grassroots *frugal innovators* with the underlying science including *frugal design* methodology.

The systematic realization of *frugal* products warrants the employment of qualified workers with a good grasp of the fundamentals of science and engineering. The sophisticated nature of innovations listed in [1,7] attests to the necessity of ‘intelligent’ designing of *frugal innovations* so that functionality is not compromised under a wide range of working conditions due to a no-frills streamlined structure. In fact, the development of these innovations should be grouped under research activities, and cutting-edge research should be employed where needed to create myriads of *frugal innovations*. In particular, *research and development* activities should be focused not least on the effective application of the *frugal design* approach; search for quantifying novel material-saving schemes; and employing cutting-edge areas such as *artificial intelligence* (AI) to strengthen the *frugal* approach due to its iterative nature showcased in figure 1.

## 7. Conclusion

It is imperative from here onwards to use *frugality*, which had its origins in the ingenuity of grassroots innovators, as an important tool of *sustainability* for saving Earth's resources and also improving living standards of society at large. However, this effort has expounded on the success of *frugal* products from here onwards hinging on the stringent application of scientific principles. In particular, the weaknesses and strengths of *frugal* products in general have been demonstrated through the example of a shaft, which is a workhorse of engineering. A tighter *factor of safety* in *classical* design has been shown to use lesser amounts of material resources while sacrificing the shaft's ability to withstand some overloading. A low *safety factor* could also impair the functioning of *frugal* products in general under nominal working conditions. Consequently, *classical* design involving a low *factor of safety* has been used to advantage in a recently developed *frugal* approach that uses the *factor of frugality*, a modern extension of the *safety factor*. The significant potential of the *frugal* approach in general has been demonstrated by the improvement of the *factor of frugality* by 167% vis-à-vis the *classical* approach while designing a simple shaft for transmitting power under pure torsional loads. Therefore, the *factor of frugality* can systematically transform an arbitrary design into a *frugal* product possessing beneficial features such as streamlined design, maximum material savings, robust functionality and low cost. Other than AFIs, the use of *frugality factor* for systematically designing grassroots innovations would further unlock their potential for widespread *sustainable development*.

## Data accessibility

This work does not have any experimental data.
